# A *Physarum* Centrality Measure of the Human Brain Network

**DOI:** 10.1038/s41598-019-42322-7

**Published:** 2019-04-11

**Authors:** Hunki Kwon, Yong-Ho Choi, Jong-Min Lee

**Affiliations:** 1 Department of Biomedical Engineering, Hanyang University, Seoul, South Korea; 2 Department of Neurology, Yale University School of Medicine, New Haven, Connecticut USA

## Abstract

The most important goals of brain network analyses are to (a) detect pivotal regions and connections that contribute to disproportionate communication flow, (b) integrate global information, and (c) increase the brain network efficiency. Most centrality measures assume that information propagates in networks with the shortest connection paths, but this assumption is not true for most real networks given that information in the brain propagates through all possible paths. This study presents a methodological pipeline for identifying influential nodes and edges in human brain networks based on the self-regulating biological concept adopted from the *Physarum* model, thereby allowing the identification of optimal paths that are independent of the stated assumption. Network hubs and bridges were investigated in structural brain networks using the *Physarum* model. The optimal paths and fluid flow were used to formulate the *Physarum* centrality measure. Most network hubs and bridges are overlapped to some extent, but those based on *Physarum* centrality contain local and global information in the superior frontal, anterior cingulate, middle temporal gyrus, and precuneus regions. This approach also reduced individual variation. Our results suggest that the *Physarum* centrality presents a trade-off between the degree and betweenness centrality measures.

## Introduction

The human brain is considered as a complex network that integrates structural and functional information^1^. A graph theoretical approach allows the quantitative analysis of the human brain based on *in vivo* brain imaging data, and can be used to increase our understanding of how brain regions are interconnected in networks^1–3^.

One of the most important goals of the brain network analysis is to detect pivotal regions and connections that strongly contribute to disproportionate communication flow to integrate global information and make the brain network more efficient^4,5^. These pivotal regions and connections are usually defined as network “hubs” at the nodal level, and network “bridges” at the edge level, that can efficiently translate signals from other brain regions along short communication paths^4–8^. Several human brain lesion studies have provided evidence that specific brain regions or bridges related to vital neurocognitive functions could be considered as candidate hubs or bridges^9–11^. These properties have been also described in several other mammalian species, such as macaques^12,13^ and cats^14^, thereby suggesting that common patterns of construction are shared across various species. Many studies have focused on brain network hubs and bridges to investigate how a disease spreads in a network and how these relate to clinical brain disorders^3,5,15,16^. It is known that the loss of hubs or bridges could reduce the effective information flow through the brain network^17–20^.

Many previous studies have identified brain network hubs and bridges using various local measures, such as the degree and betweenness centralities^8,9,21–27^. Therefore, it is important to interpret their roles in the network according to the measures used in the study^3,5^. The degree centrality (***C***_***D***_) is defined as the number of edges connected to a node, is an extensively adopted measure used to quantify the local centrality of each node, and has a direct neurobiological interpretation^3,28^. Unlike the degree of a node, which is regarded as a local part of centrality, some centrality measures represent the importance of a node based on the concept of the shortest path between any two nodes in the brain network^28–30^. Betweenness centrality (***C***_***B***_) is calculated as the fraction of the number of the shortest paths that pass through a given node or edge to the total number of shortest paths, and has been extensively used^30,31^. A node or an edge with an increased ***C***_***B***_ value indicates a large influence on the transfer of information across brain regions.

It is noted that most centrality measures commonly used in brain network analyses assume that the information flow in a network propagates only through the single shortest path. However, this assumption is not true for most real networks^32–34^. For example, traffic will likely follow alternative paths if the shortest path is congested, and information about computer viruses, news, rumors, or infections, will likely propagate through random paths in a network, rather than through the single shortest path^35^. The mechanisms of how the network communication flows in the brain remain unclear, but it has been suggested that the information in the brain naturally propagates along all possible paths, and not only through the shortest paths^36–38^. Recently, some brain network studies have attempted to address the shortest path assumption. These studies have used a maximum-flow-inspired algorithm (which constrains paths between regions using a flow-connectivity matrix), instead of the shortest paths between two regions, to measure the flow between network regions^39,40^. These studies did not investigate changes of network hubs or bridges in accordance with the shortest path assumption, but focused instead on the understanding of the information flow between network regions as new properties.

Nakagaki *et al*. suggested a self-regulating biological model using the amoeboid organism “*Physarum polycephalum*,” to identify (a) optimal paths to connect two food sources by controlling the amount of fluid flow, and (b) competing paths in a tubular network^41,42^. This model has been successfully applied in various fields to solve the optimization, shortest path, and the 0–1 knapsack problems^43–47^. In particular, a bio-inspired network local measure called “*Physarum* centrality (***C***_***P***_)” has been suggested to identify the centrality of brain regions over the network by combining the fluxes of the edges linked to specific nodes^32,45^. Because the information flow in the brain has been transmitted not only through the shortest paths but also through many connected paths, ***C***_***P***_ could be suitable for extracting the important regions and for identifying the connections in a brain network. However, to the best of our knowledge, ***C***_***P***_ has not been previously applied in brain network analyses.

This study aimed to identify the influential nodes and edges of human brain networks based on ***C***_***P***_. In addition, we compared the ***C***_***P***_ results with those based on commonly used network centrality measurements, such as ***C***_***D***_ and ***C***_***B***_, to examine the effect of the shortest path assumption.

## Results

### Spatial distribution of hub nodes and bridge edges

**Figure 1:**
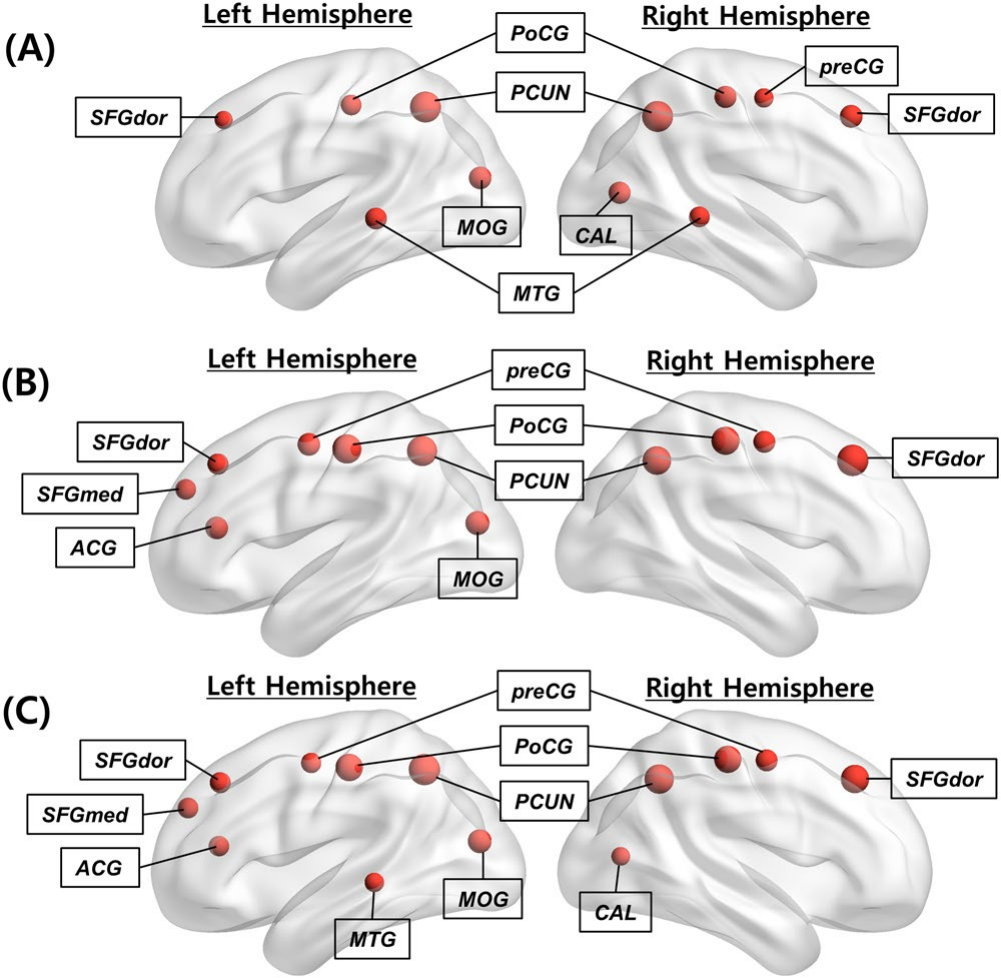
Distribution of network hub nodes based on ***C***_***D***,***node***_, ***C***_***B***,***node***_ and ***C***_***P***,***node***_. (**A**) Network hub nodes based on ***C***_***D***,***node***_ are highlighted by the red circles. (**B**) Network hub nodes based on ***C***_***B***,***node***_ are highlighted by the red circles. (**C**) Network hub nodes based on ***C***_***P***,***node***_ are highlighted by the red circles. The network hub nodes were identified when the network nodes were greater than one standard deviation (SD) above the mean of each nodal centrality measure map. The size of each circle indicates the strength of each centrality measure.

**Figure 2:**
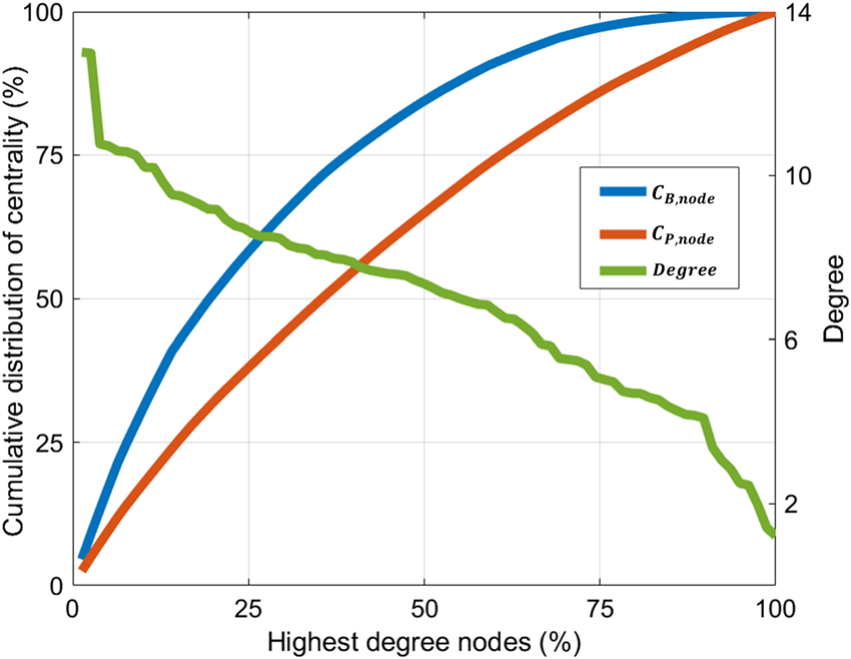
Cumulative distributions of ***C***_***B***,***node***_ and ***C***_***P***,***node***_with degree. Nodes were sorted so that the node with the highest value moved to one, and the node with the lowest centrality value moved to the last index (x–axis). The cumulative distribution of ***C***_***B***,***node***_ is shown in blue, the cumulative distribution of ***C***_***P***,***node***_ is shown in red, and the degree distribution is shown in green.

**Figure 3:**
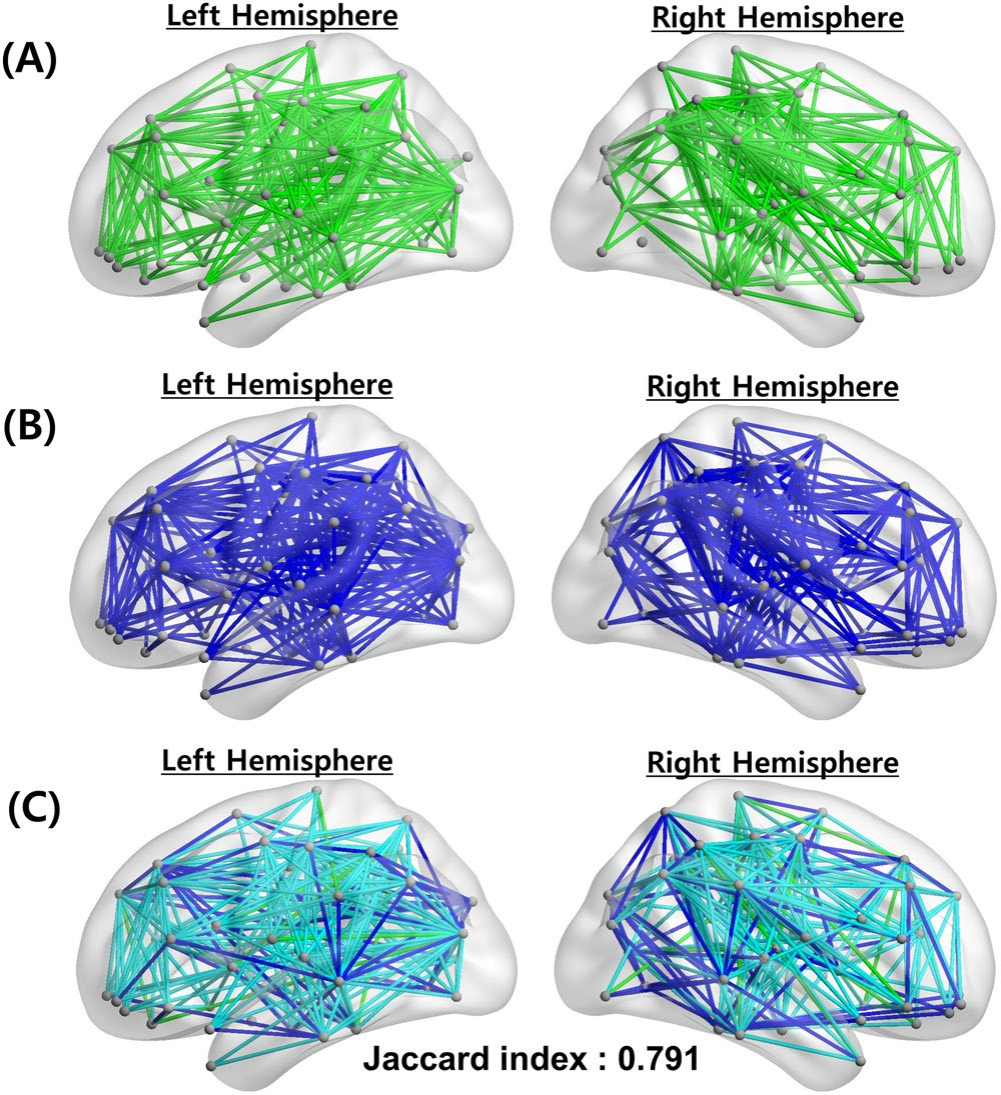
Distribution of network bridge edges based on ***C***_***B***,***edge***_ and ***C***_***P***,***edge***_. (**A**) Network bridge edges based on ***C***_***B***,***edge***_ are shown in green. (**B**) Network bridge edges based on ***C***_***P***,***edge***_ are shown in blue lines. (**C**) Overlapped bridge edges between ***C***_***B***,***edge***_ and ***C***_***P***,***edge***_ are shown in light blue lines. The network bridge edges are identified when the network edges are greater than one standard deviation (SD) above the mean of each edge centrality measure map. Their Jaccard index is also shown with overlapped bridge edges.

The network hubs of three centrality measures (one standard deviation above the mean), i.e., ***H***(***C***_***D***,***node***_), ***H***(***C***_***B***,***node***_), and ***H***(***C***_***P***,***node***_), were identified according to each centrality map, i.e., ***C***_***D***,***node***_, ***C***_***B***,***node***_, and ***C***_***P***,***node***_. It is noted that the anatomical locations of the obtained hubs were adopted from the predefined template^48^ (Table S1). Accordingly, the ***H***(***C***_***D***,***node***_) measure appeared in four cortical regions (the precuneus, middle temporal gyrus, superior frontal gyrus [dorsolateral], and postcentral gyrus) in a bilaterally symmetric fashion, and in three other regions (the right precentral gyrus, right calcarine fissure, and left middle occipital gyrus) (Fig. 1A). Furthermore, ***H***(***C***_***B***,***node***_) appeared in four cortical regions (the precuneus, superior frontal gyrus [dorsolateral], precentral gyrus, and postcentral gyrus) in a bilaterally symmetric fashion, as well as in three other regions (the left superior frontal gyrus [medial], left anterior cingulate and paracingulate gyrus, and left middle occipital gyrus) (Fig. 1B). Equivalently, ***H***(***C***_***P***,***node***_) appeared in nine cortical regions (the precuneus, superior frontal gyrus [dorsolateral], precentral gyrus, postcentral gyrus, left superior frontal gyrus [medial], right calcarine fissure, left middle temporal gyrus, left middle occipital gyrus, and in the left anterior cingulate and paracingulate gyri) (Fig. 1C). Figure 2 shows the cumulative distributions of ***C***_***B***,***node***_ and ***C***_***P***,***node***_ with a degree distribution in a continuous manner. Accordingly, ***C***_***B***,***node***_ and ***C***_***P***,***node***_ accounted for 83.47% and 63.67% of the top 50% most connected nodes, respectively. The results indicate that ***C***_***P***,***node***_ is homogenously distributed compared to ***C***_***B***,***node***_, and the extensive diversity in the paths from the *Physarum* model can improve efficiency, robustness, and the resilience of the brain network^49,50^. We also investigated how the network bridges (one standard deviation above the mean), i.e., ***B***(***C***_***B***,***edge***_) and ***B***(***C***_***P***,***edge***_), according to the edge betweenness centrality (***C***_***B***,***edge***_) and edge *Physarum* centrality (***C***_***P***,***edge***_) distribute (Fig. 3). Additionally, ***C***_***B***,***edge***_ was calculated as the number of the shortest paths that pass through a given edge, and ***C***_***P***,***edge***_ was calculated by the sum of the flux that passes through a given edge from the *Physarum* model (see Methods).

### Overlap of hub nodes and bridge edges

**Figure 4:**
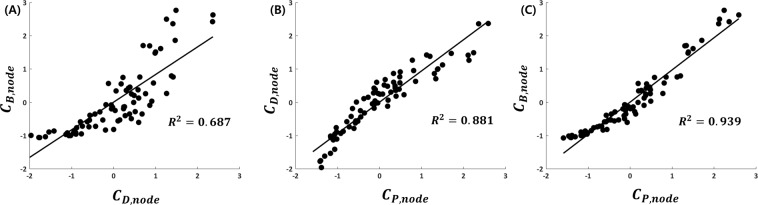
Scatter plots of centrality measures with correlation lines. Each centrality is normalized by subtracting the mean and then dividing the standard deviation to allow unbiased comparisons. There are significant positive correlations for three different pairs: (**A**) ***C***_***D***,***node***_ vs. ***C***_***B***,***node***_, (**B**) ***C***_***P***,***node***_ vs. ***C***_***D***,***node***_, and (**C**) ***C***_***P***,***node***_ vs. ***C***_***B***,***node***_. Each circle represents a node, and the black line represents a correlation line.

The Jaccard indices (***J***) for each pair of network hub sets (***H***(***C***_***D***,***node***_) vs. ***H***(***C***_***B***,***node***_), ***H***(***C***_***P***,***node***_) vs. ***H***(***C***_***D***,***node***_), and ***H***(***C***_***P***,***node***_) vs. ***H***(***C***_***B***,***node***_)) were estimated (Table 1). ***J***(***H***(***C***_***P***,***node***_), ***H***(***C***_***B***,***node***_)) had the highest value (0.846), and ***J***(***H***(***C***_***D***,***node***_), ***H***(***C***_***B***,***node***_)) had the lowest value (0.571). Linear regression analyses were performed for three pairs of centrality measures (Fig. 4). We found that ***C***_***P***,***node***_ was positively correlated with ***C***_***B***,***node***_ (*R*^2^ = 0.939, *P* = 1.8606*e*^−48^), and ***C***_***D***,***node***_ was positively correlated with ***C***_***B***,***node***_ (*R*^2^ = 0.687, *P* = 2.6774*e*^−21^). It was noted that the Jaccard index and regression analysis, including ***C***_***P***,***node***_, exhibited a strong tendency to acquire higher values compared to the values of other models. Notably, most of the network hub regions overlapped to some extent. In all three centrality measures, three cortical regions (the precuneus, superior frontal gyrus, and postcentral gyrus) appeared in a bilaterally symmetric fashion, while two regions (the left middle occipital gyrus and right precentral gyrus) appeared in a lateralized manner. We also calculated ***J***(***B***(***C***_***B***,***edge***_), ***B***(***C***_***P***,***edge***_)) on the edge level to discover common efficient communication paths between two different measures. Its value was estimated to equal 0.791. Notably, the results suggest that the overlapped paths may be core paths, and they may have an important role in the efficient information flow across brain regions.

**Table 1:** Jaccard indices between network hubs from three centrality measures.

	*H* (*C*_*D,node*_)	*H* (*C*_*B,node*_)	*H* (*C*_*P,node*_)
***H*** (***C***_***D****,****node***_)	1	0.571	0.714
***H*** (***C***_***B****,****node***_)	0.571	1	0.846
***H*** (***C***_***P****,****node***_)	0.714	0.846	1

The Jaccard index of the hub regions is the ratio of the number of overlapping hub nodes to the total number of hub nodes based on any two centrality measures. The value of the Jaccard index varies from zero (no overlap) to one (perfect overlap).

### Differences of hub nodes and bridge edges

A post-hoc analysis was performed after the analysis of variance (ANOVA) test on the network hub regions based on three Z-transformed centrality measures (***C***_***D***,***node***_ vs. ***C***_***B***,***node***_ vs. ***C***_***P***,***node***_). Table 2 shows the details of the ANOVA and post-hoc analyses. In the five cortical hub regions (the left precentral gyrus, right superior frontal gyrus [dorsolateral part], left anterior cingulate, and left and right postcentral gyri), ***C***_***D***,***node***_ was significantly lower than ***C***_***B***,***node***_ a ***C***_***P***,***node***_, but ***C***_***B***,***node***_ and ***C***_***P***,***node***_ were not significantly different. In the three cortical hub regions (the right precentral gyrus and left superior frontal gyrus [dorsolateral and medial part]), only ***C***_***P***,***node***_ was significantly higher than ***C***_***D***,***node***_. In the four cortical hub regions (the calcarine fissure, right precuneus, and left and right middle temporal gyri), ***C***_***D***,***node***_ was significantly higher than ***C***_***B***,***node***_and ***C***_***P***,***node***_, and ***C***_***P***,***node***_was significantly higher than ***C***_***B***,***node***_ **(*C***_***D***,***node***_ > ***C***_***P***,***node***_ > ***C***_***B***,***node***_**)**. A similar tendency was observed in the left precuneus. Table 3 shows the differences of the bridge edges based on the ***C***_***B***,***edge***_ and ***C***_***P***,***edge***_ values. ***B***(***C***_***P***,***edge***_) contained 46 additional bridge edges which ***B***(***C***_***B***,***edge***_) did not have. The additional ***B***(***C***_***P***,***edge***_) mainly connected with hub nodes, such as the calcarine fissure and the middle temporal gyrus, rather than ***B***(***C***_***B***,***edge***_). However, ***B***(***C***_***B***,***edge***_) had only 18 additional bridge edges which ***B***(***C***_***P***,***edge***_) did not have (Table 4**)**.

**Table 2:** Comparison of three centrality measures of all hub regions.

Hub regions	F–value	P–value	*C*_*D*,*node*_ (Mean ± SD)	*C*_*B*,*node*_ (Mean ± SD)	*C*_*P*,*node*_ (Mean ± SD)	Post-hoc test
PreCG.L	18.335	<0.0001^†^	0.746 ± 0.027	1.148 ± 0.063	1.021 ± 0.047	***C***_***D***,***node***_ < ***C***_***B***,***node***_, ***C***_***D***,***node***_ < ***C***_***P***,***node***_
PreCG.R	4.421	0.012^†^	0.977 ± 0.026	1.085 ± 0.061	1.172 ± 0.045	***C***_***D***,***node***_ < ***C***_***P***,***node***_
SFGdor.L	5.04	0.007^†^	0.88 ± 0.026	1.009 ± 0.057	1.075 ± 0.044	***C***_***D***,***node***_ < ***C***_***P***,***node***_
SFGdor.R	27.296	<0.0001^†^	1.308 ± 0.028	1.845 ± 0.073	1.733 ± 0.053	***C***_***D***,***node***_ < ***C***_***B***,***node***_, ***C***_***D***,***node***_ < ***C***_***P***,***node***_
SFGmed.L	5.25	0.005^†^	0.869 ± 0.029	1.009 ± 0.061	1.083 ± 0.047	***C***_***D***,***node***_ < ***C***_***P***,***node***_
ACG.L	30.478	<0.0001^†^	0.625 ± 0.027	1.17 ± 0.069	1.057 ± 0.052	***C***_***D***,***node***_ < ***C***_***B***,***node***_, ***C***_***D***,***node***_ < ***C***_***P***,***node***_
CAL.R	84.506	<0.0001^†^	1.242 ± 0.027	0.528 ± 0.048	0.885 ± 0.039	***C***_***D***,***node***_ > ***C***_***P***,***node***_ > ***C***_***B***,***node***_
MOG.L	0.653	0.521	1.297 ± 0.031	1.25 ± 0.07	1.337 ± 0.053	
PoCG.L	32.036	<0.0001^†^	1.098 ± 0.029	1.701 ± 0.079	1.673 ± 0.061	***C***_***D***,***node***_ < ***C***_***B***,***node***_, ***C***_***D***,***node***_ < ***C***_***P***,***node***_
PoCG.R	18.087	<0.0001^†^	1.238 ± 0.027	1.606 ± 0.071	1.653 ± 0.053	***C***_***D***,***node***_ < ***C***_***B***,***node***_, ***C***_***D***,***node***_ < ***C***_***P***,***node***_
PCUN.L	8.408	<0.0001^†^	2.083 ± 0.024	1.776 ± 0.078	2.028 ± 0.055	***C***_***D***,***node***_ > ***C***_***B***,***node***_, ***C***_***B***,***node***_ < ***C***_***P***,***node***_
PCUN.R	19.892	<0.0001^†^	2.074 ± 0.025	1.626 ± 0.068	1.842 ± 0.048	***C***_***D***,***node***_ > ***C***_***P***,***node***_ > ***C***_***B***_,_***node***_
MTG.L	48.873	<0.0001^†^	1.217 ± 0.029	0.544 ± 0.06	0.948 ± 0.051	***C***_***D***,***node***_ > ***C***_***P***,***node***_ > ***C***_***B***,***node***_
MTG.R	156.231	<0.0001^†^	1.113 ± 0.024	0.191 ± 0.045	0.622 ± 0.038	***C***_***D***_,_***node***_ > ***C***_***P***_,_***node***_ > ***C***_***B***_,_***node***_

An ANOVA test was performed to determine significant differences among Z-transformed centrality measures (***C***_***D***,***node***_, ***C***_***B***,***node***_, and ***C***_***P***,***node***_) at all 78 network nodes. Values of P < 0.05 were accepted as significant with Bonferroni post-hoc correction. ^†^FDR corrected P < 0.05.

**Table 3:** Network bridge edges based on Physarum centrality.

*Region 1*	*Region 2*	*C*_*P,edge*_	*C*_*B,edge*_
SFGmed.L	CUN.L	1.767	0.906
SOG.L	MTG.L	1.587	0.880
SFGmed.L	DCG.L	1.562	0.909
LING.L	MTG.L	1.486	0.862
SFGdor.L	INS.L	1.427	0.844
MOG.L	IPL.L	1.396	0.975
MOG.L	SPG.L	1.390	0.861
ANG.L	MTG.L	1.381	0.722
CUN.L	MOG.L	1.327	0.869
SFGdor.L	FFG.L	1.312	0.929
PHG.L	MTG.L	1.311	0.813
CAL.R	STG.R	1.300	0.841
DCG.R	PCUN.R	1.290	0.832
PCUN.L	DCG.R	1.276	0.856
SFGdor.L	SOG.L	1.269	0.844
SFGdor.L	MOG.L	1.263	0.963
SMG.L	MTG.L	1.258	0.709
PreCG.R	PCUN.R	1.221	0.824
PoCG.L	MTG.L	1.218	0.639
ACG.L	SOG.L	1.216	0.809
PCUN.L	SMA.R	1.205	0.666
PreCG.R	MFG.R	1.201	0.910
PCUN.L	PCUN.R	1.200	0.791
DCG.L	PCUN.R	1.196	0.811
IFGtriang.L	MTG.L	1.171	0.817
PCG.R	PCUN.R	1.166	0.827
SFGdor.L	ITG.L	1.163	0.782
SFGdor.R	SFGmed.R	1.132	0.817
PreCG.L	SOG.L	1.129	0.907
MOG.L	MTG.L	1.117	0.480
PreCG.R	MTG.R	1.087	0.670
SFGdor.R	ITG.R	1.087	0.796
ACG.L	LING.L	1.086	0.533
DCG.R	CAL.R	1.081	0.546
PreCG.L	IPL.L	1.064	0.660
SFGdor.R	CUN.R	1.061	0.684
PreCG.R	INS.R	1.059	0.937
SFGmed.L	CAL.L	1.052	0.469
SOG.L	PoCG.L	1.050	0.984
SFGdor.R	PoCG.R	1.044	0.983
SFGdor.L	SFGmed.L	1.036	0.491
SFGdor.L	SMA.L	1.023	0.483
PreCG.L	MFG.L	1.018	0.621
SOG.R	PoCG.R	1.017	0.920
ACG.L	FFG.L	1.013	0.660
SFGdor.L	IOG.L	1.002	0.764

Forty-six network bridges ***C***_***P***,***edge***_ are listed in a descending order of normalized ***C***_***P***,***edge***_ values based only on the edge *Physarum* centrality (***C***_***P***,***edge***_) values. Network bridges are defined as edges when ***C***_***P***,***edge***_ is greater by one standard deviation above the mean. Normalized edge betweenness centrality (***C***_***B***,***edge***_) is also listed on the same connection label.

**Table 4:** Network bridge edges based on betweenness centrality.

*Region 1*	*Region 2*	*C*_*B,edge*_	*C*_*P,edge*_
PreCG.L	SMG.R	1.535	0.903
MOG.L	ANG.R	1.531	0.962
SFGmed.L	INS.R	1.393	0.933
SFGdor.L	PreCG.R	1.380	0.821
IFGoperc.L	PCUN.L	1.370	0.866
CAL.R	HES.R	1.365	0.735
PreCG.L	DCG.R	1.253	0.957
ROL.L	MOG.L	1.227	0.957
REC.L	MOG.L	1.194	0.723
MOG.L	MTG.R	1.136	0.821
SFGmed.L	REC.L	1.082	0.768
PreCG.R	HES.R	1.073	0.857
ORBinf.R	PoCG.R	1.070	0.783
SFGdor.R	OLF.R	1.036	0.393
ROL.R	PCUN.R	1.029	0.559
SFGdor.R	PCL.R	1.016	0.862
FFG.L	PCUN.L	1.015	0.962
PCG.L	PoCG.R	1.007	0.640

Eighteen network bridges ***C***_***B***,***edge***_are listed in a descending order of normalized ***C***_***B***,***edge***_ values based only on the edge betweenness centrality (***C***_***B***,***edge***_). Network bridges are defined as edges when ***C***_***B***,***edge***_ is greater by one standard deviation above the mean. The normalized edge *Physarum* centrality (***C***_***P***,***edge***_) is also listed on the same connection label.

### Individual variability of hub nodes

The coefficient of variation (***CV***) was calculated in network hub regions, including the left and right precentral gyri, left and right superior frontal gyri (dorsolateral part), left superior frontal gyrus (medial part), left anterior cingulate, left calcarine fissure, left middle occipital gyrus, left and right postcentral gyri, left and right precuneus, and left and right middle temporal gyri (Table 5). A two-tailed *t*-test was performed to determine the statistical significance of the differences in ***CV*** values among the three centrality measures (***C***_***D***,***node***_, ***C***_***B***,***node***_ and ***C***_***P***,***node***_). Accordingly, it was found that ***CV*** (***C***_***D***,***node***_) was significantly lower than ***CV*** (***C***_***B***,***node***_) (*P* = 2.5610*e*^−19^, *t*-test) and ***CV***(***C***_***P***,***node***_) (*P* = 1.2673*e*^−12^, *t*-test). Additionally, ***CV***(***C***_***P***,***node***_) was also significantly lower than ***CV***(***C***_***B***,***node***_) (*P* = 8.2899*e*^−18^, *t*-test). Furthermore, the values of ***CV***(***C***_***B***,***node***_), ***CV***(***C***_***D***,***node***_), and ***CV***(***C***_***P***,***node***_) were found to lie in the ranges of 0.5777–0.6776, 0.1478–0.1778, and 0.2378–0.3138, respectively.

**Table 5:** Coefficients of variation (CV) in the network hub regions of three centrality measures.

Hub regions	*C*_*D*, *node*_	*C*_*B*, *node*_	*C*_*P*, *node*_
PreCG.L	—	0.6394	0.2845
PreCG.R	0.1624	0.6424	0.2655
SFGdor.L	0.1578	0.6276	0.2693
SFGdor.R	0.1524	0.5777	0.2828
SFGmed.L	—	0.6600	0.2831
ACG.L	—	0.6776	0.3068
CAL.R	0.1588	—	0.2462
MOG.L	0.1690	0.6766	0.2981
PoCG.L	0.1778	0.6195	0.3059
PoCG.R	0.1568	0.5830	0.2715
PCUN.L	0.1231	0.5839	0.2538
PCUN.R	0.1208	0.5595	0.2378
MTG.L	0.1635	—	0.3138
MTG.R	0.1478	—	—

The coefficient of variation was quantified as a measure of intersubject variability. A lower CV value indicates lower intersubject variability and a higher consistency across subjects in the group.

## Discussion

In this study, we proposed a novel methodological framework for defining the importance of network nodes and edges using the *Physarum* model. Other centrality measures, such as ***C***_***D***_ and ***C***_***B***_, assume that information flows in a network only through the paths that are associated with the shortest connections, but ***C***_***P***_ considers all possible information flows between brain regions.

Many previous studies have detected brain network hubs and bridges using various measures, such as ***C***_***D***_ and ***C***_***B***_^8,9,21–27,51^. These measures of centrality helped the interpretation of the meaning of nodes and edges in the network^3,5^. Accordingly, ***C***_***D***,***node***_, usually defined as the number of connections of the target node, quantified the local properties without global information flow. Furthermore, ***C***_***B***,***node***_ identified the node that played an important role with the use of information based on the global flow patterns and on the shortest path concept, while ***C***_***B***,***edge***_ used a similar approach to that used by ***C***_***B***,***node***_ at the edge level. Equivalently, ***C***_***P***_ and ***C***_***B***_ used the global information flow. However, ***C***_***P***_ used all the possible paths from the *Physarum* model instead of the shortest path concept and was shown to be affected by local characteristics, such as ***C***_***D***_.

As shown in Fig. 2, the ***C***_***P***,***node***_ was homogenously distributed among all network nodes compared to ***C***_***B***,***node***_. However, the ***C***_***P***,***node***_ considered the optimal paths from the *Physarum* model independent of the assumption used by other centralities according to which the information flow of a network only spread through the shortest connecting paths. Previous studies have shown the existence of a communication scheme that contradicted the assumption that only the shortest connections are used^37,52^. These studies have shown that ***C***_***P***_ can be uniformly distributed compared to ***C***_***B***_.

The Jaccard index was used to examine the overlap ratio between the sets of each centrality measure. As shown in Table 1, the Jaccard indices between hub sets based on ***C***_***P***,***node***_ and ***C***_***B***,***node***_ had higher values than those associated with other combinations. As shown in Fig. 4, similar Jaccard index patterns were observed between ***C***_***P***,***node***_ and other measures in a continuous manner. The Jaccard index value estimated between bridge sets based on ***C***_***P***,***edge***_ and ***C***_***B***,***edge***_ also yielded higher values. Thus, the network hub regions determined based on ***C***_***P***,***node***_ possessed local and global network properties. Based on global information, some regions of the precentral gyrus and superior frontal and anterior cingulate gyri were defined as network hubs. However, these regions were not considered as network hubs based on local information, such as ***C***_***D***,***node***_. In previous studies, these regions were classified as multimodal and functional hubs that are parts of cognitive resting-state networks, such as the default mode^5,25^. In addition, these regions were also defined as network hubs in other species, like in macaques and cats^14,22,53^. Some studies have found that the high FA values in the superior frontal gyrus were associated with post-traumatic stress disorder^54^, and exhibited decreased blood oxygen level-dependent activation of the superior frontal gyrus during a working memory task in individuals with schizotypal personality disorders^55^. FA plays an important role in the detection of network hub regions in global communication processes in the superior frontal and anterior cingulate regions^21^. As shown in Table 2, ***C***_***D***,***node***_ has lower values than ***C***_***B***,***node***_ and ***C***_***P***,***node***_ in the superior frontal and anterior cingulate regions.

Although similar patterns were observed in network hubs (Table 1 and Fig. 1) and bridges (Fig. 3) based on ***C***_***B***_ and ***C***_***P***_ because they both used global information, and because their core paths exhibited similar patterns, some regions, such as the calcarine fissure and the middle temporal gyrus, could not be detected based on ***C***_***B***,***node***_, which measures only the shortest path between network regions. Notably, the middle temporal gyrus is a meaningful network hub^25,56^. The association of the middle temporal gyrus is reduced on voxel-based DTI measures^57^, and network efficiency and centrality in the middle temporal gyrus have been shown to be disrupted in individuals with Alzheimer’s disease^58,59^. The grey matter volume is reduced in the middle temporal gyrus in individuals with schizotypal personality disorders^60,61^. The bridges based on ***C***_***P***,***edge***_ (Table 3) also yielded more connections with the calcarine fissure and the middle temporal gyrus compared to the bridges based on ***C***_***B***,***edge***_ (Table 4). The precuneus plays an important role in the brain network, thus suggesting that it has mutual connections with other areas^56,62^. Specifically, the precuneus was connected with parietal regions that were related to visuo–spatial information processing^63^. Both ***C***_***B***,***node***_ and ***C***_***P***,***node***_ could detect the precuneus as a network hub (Fig. 1B,C). However, network hubs based on ***C***_***P***,***node***_ reflected the important network properties of the precuneus in a better manner compared to ***C***_***B***,***node***_ (Table 2). Additionally, network bridges based on ***C***_***P***,***edge***_ also included more connections with precuneus than ***C***_***B***,***edge***_ (Table 3).

The process of competition to find the optimal paths—instead of the shortest paths—from the *Physarum* model required increased information flow. Accordingly, it would be helpful to enhance the flow information efficiently across different brain regions. Centrality measures identified based on the shortest path assumption have been used in many brain network analyses, such as computer viruses, news, rumors, or infections^32–35^, but they have not been used in most real networks. In the brain network, there are some considerations against the shortest path assumption because it is difficult to elucidate the mechanism of an action potential that encodes the route and its destinations^52^. The shortest path assumption can also lead to nonresilient communication or loss of information^52,64^. Accordingly, the communication model with multiple connected paths is likely to be more appropriate, and can produce various alternative paths, which increase the efficiency, robustness, and resilience of the brain network^50,65^. The *Physarum* model has been suggested to combine the flux of tubular networks and competing edges through many possible paths. Therefore, we conclude that the *Physarum* model can improve the efficiency, robustness, and resilience of the brain network.

It is important to investigate the common features and variability of network centralities across subjects, and it is also critical to minimize the intrasubject variability^66,67^. The coefficient of variation (***CV***) is computed to describe the variation across subjects. As shown in Table 5, the ***CV*** of ***C***_***P***,***node***_ was significantly lower than that of ***C***_***B***,***node***_. This indicated that the *Physarum* network hubs were more consistent throughout the dataset. Notably, network hubs based on ***C***_***D***,***node***_ yielded the lowest ***CV*** values compared to those based on centrality measures. This is because ***C***_***D***,***node***_ is a relatively simple method, and local information is less variable than global information. Therefore, ***C***_***P***,***node***_ may capture the characteristics of local information to detect network hubs.

Many previous studies on brain network analyses have used various predefined atlases to define network nodes. The choice of the atlas defining the network nodes affects the network measures^66^. The spatial location of highly connected brain regions can be different depending on which atlas is used^66,68^. While the automated anatomical labeling (AAL) atlas was used in this study to compare and interpret the existing network hub and bridge results obtained in the previous studies^8,25,69^, some rigorous experiments with different atlases will be needed in future studies to compare the effects of atlas selection.

In this study, we illustrated a novel methodological framework for the identification of influential nodes and connections of the human brain network based on ***C***_***P***_. This model has not been previously employed in a brain network. Comparison of the validation results between ***C***_***P***_ and other network centrality measurements indicated that ***C***_***P***_ contained local and global information. Additionally, this measure was not based on the assumption that the information flow of a network spread only through the shortest connections. Accordingly, ***C***_***P***_ could reduce the within-individual variation and detect some regions and connections that are related to post-traumatic stress disorder, schizotypal personality disorder, and Alzheimer’s disease. Therefore, it would be helpful to apply this measure to individuals with neurological disorders that could provide biologically meaningful network results.

## Methods

### Subjects and data acquisition

This study used the Human Connectome Project (HCP, https://www.humanconnectome.org/) dataset and included 339 healthy participants (age: 28.2 ± 3.9 years, female: 159, male: 180). Their scans and data were released after they passed the HCP quality control and assurance standards^70^. Table 6 shows the details of these datasets.

**Table 6:** Demographic information of participants.

	Total	Male	Female
Number of subjects	307	146	161
Age (mean ± SD) (years)	28.45 ± 3.65	28.23 ± 3.58	28.65 ± 3.70

### Data preprocessing

An automated processing-pipeline (CIVET) was used to process T_1_-weighted magnetic resonance (MR) images (http://mcin-cnim.ca/neuroimagingtechnologies/civet/)^71^. The T_1_-weighted MR images were first registered to ICBM152 T1 template in the Montreal Neurological Institute (MNI) space using an affine linear transformation^72^, and were then corrected for intensity nonuniformities owing to magnetic field inhomogeneities using an N3 algorithm^73^. After the removal of tissues unrelated to the brain matter, registered and corrected images were segmented into the white matter, grey matter, cerebrospinal fluid, and background, using an advanced neural-net classifier^71^.

Diffusion Tensor Imaging (DTI) datasets were managed using the FMRIB’s software library (http://www.fmrib.ox.ac.uk/fsl). Motion artifacts and eddy current distortions were corrected by normalizing diffusion-weighted images to the baseline image using the affine registration in the FMRIB’s linear image registration tool (FLIRT). A diffusion tensor matrix from the corrected diffusion-weighted images was generated based on a simple linear fitting algorithm, and the FA of each voxel was then calculated. DTI tractography was performed in the diffusion MR space using the FACT algorithm^74^, and was implemented using the Diffusion Toolkit (http://trackvis.org/) for the extraction of approximately 100,000 fibers from each subject. An angle of <45° between each fiber tracking step and a minimum/maximum path length of 20/200 mm were set as the terminating conditions. The classified white matter map masked the tractography results to eliminate false positives.

### Construction of structural connectivity matrices

**Figure 5:**
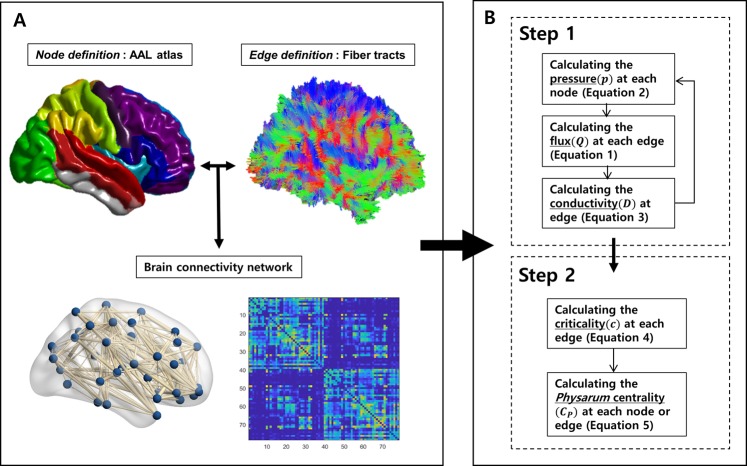
Flowchart of measurement of Physarum centrality. The process for Physarum centrality (***C***_***P***_) measurement was assessed in two steps. In step 1, the optimal path using the Physarum model was iteratively calculated within all pairs of network nodes, respectively. In step 2, ***C***_***P***_ was extracted in each node or edge based on the optimal path within all pairs of network nodes.

It is important to define the basic elements of networks as edges. Because definitions and processes of constructing network nodes and edges have been described in detail previously, we explained them briefly as follows^69,75,76^ (Fig. 5A).

#### Node definition

We used the AAL atlas^48^ with the exception of the cerebellum and subcortical regions to segment the cortical regions into 78 areas, which represent the nodes of the network. Individual T_1_-weighted images were nonlinearly transformed to the ICBM 152 template, and the AAL atlas in the MNI space was then transformed to the T_1_ native space using the inverse transformation parameters. Therefore, the individual AAL atlas was defined in the T_1_ native space.

#### Edge definition

Tractography results were used to quantify edges between different AAL regions for individual networks. Individual T_1_-weighted images were coregistered to the baseline image using the affine registration in the FMRIB’s FLIRT. Tractography results were transformed into the T_1_ native space using the inverse transformation parameters. Fiber tractography results and the AAL atlas thus represented the same individual T_1_ native space. Two nodes were considered to be structurally connected when at least three fiber tracts were present between these two nodes^69,75,77,78^. Accordingly, the edge was defined as the mean FA value along the fiber tracts^69,75^. Structural connectivity matrices were then constructed for each individual.

### Physarum centrality

***C***_***P***_ was calculated in two steps using an in-house software implemented in MATLAB (Version R2012b, Mathworks, Natick, MA, USA) (Fig. 5B). Based on the *Physarum* model, the optimal path was obtained within all pairs of network nodes, and ***C***_***P***_ was then calculated at each node^43,45^.

#### Physarum model for path finding

The basic concept underlying the *Physarum* model is that long and narrow tubes tend to weaken, and short and wide tubes strengthen with the positive feedback of flux in tubes during the competition process in the effort expended to identify the optimal paths. This concept assumes that short and wide tubes are the most effective for fluid transmission of information.

In a *Physarum* tubular network, each tube segment is denoted as the edge ***e***_***ij***_, and its two ends are linked nodes ***i*** and ***j***. If the flow along the tube is a Hagen−Poiseuille flow, the flux ***Q*** of each edge ***e***_***ij***_ can be defined as

1 $${{\boldsymbol{Q}}}_{{\boldsymbol{ij}}}=\frac{{{\boldsymbol{D}}}_{{\boldsymbol{ij}}}}{{{\boldsymbol{L}}}_{{\boldsymbol{ij}}}}({{\boldsymbol{p}}}_{{\boldsymbol{i}}}-{{\boldsymbol{p}}}_{{\boldsymbol{j}}}),$$

where ***p***_***i***_ and ***p***_***j***_ are the pressures at node ***i*** and ***j***, respectively. The length and width of the tubes are denoted as ***L***_***ij***_ and conductivity ***D***_***ij***_, respectively. The flux indicates the information flow, and the length and conductivity of tubes indicate the edge in the brain network. The lengths of the tubes are only calculated at the first instance, but the conductivity can be updated according to the information of flux ***Q***_***ij***_. When the characteristic magnitude of the flux from the starting node to the ending node is denoted as ***I***_0_, and the characteristic length and conductivity of the tubes are respectively denoted as $\bar{{\boldsymbol{L}}}$ and $\bar{{\boldsymbol{D}}}$, the characteristic pressure $\bar{{\boldsymbol{p}}}$ can be given by $\bar{{\boldsymbol{p}}}={{\boldsymbol{I}}}_{0}\bar{{\boldsymbol{L}}}/\bar{{\boldsymbol{D}}}$. Accordingly, the maintenance of flux through each node can be modeled as,

2 $$\sum _{{\boldsymbol{i}}}\,\frac{{{\boldsymbol{D}}}_{{\boldsymbol{ij}}}}{{{\boldsymbol{L}}}_{{\boldsymbol{ij}}}}({{\boldsymbol{p}}}_{{\boldsymbol{i}}}-{{\boldsymbol{p}}}_{{\boldsymbol{j}}})=\{\begin{array}{c}-{{\boldsymbol{I}}}_{{\bf{0}}},\,{\bf{for}}\,{\boldsymbol{j}}={\boldsymbol{s}},\\ +{{\boldsymbol{I}}}_{{\bf{0}}},\,{\bf{for}}\,{\boldsymbol{j}}={\boldsymbol{t}},\\ {\bf{0}},\,{\bf{otherwise}},\end{array}$$

where ***s*** and ***t*** are starting and ending nodes, respectively. Thus, the total flux in the brain from the starting to the ending nodes is a fixed constant ***I***_0_ during the path-finding process. Therefore, the pressure of each node and flux are calculated using Eqs (1 and 2), respectively. The flux can be updated according to the calculated pressure at all the nodes.

The conductivities of the tubes are strengthened by large fluxes based on the positive feedback in the *Physarum* model, or are weakened by small fluxes when the lengths of the tubes are maintained fixed. The conductivity ***D***_***ij***_ is thus changed over time and is expressed as,

3 $$\frac{{\boldsymbol{d}}}{{\boldsymbol{dt}}}{{\boldsymbol{D}}}_{{\boldsymbol{ij}}}={\boldsymbol{f}}(|{{\boldsymbol{Q}}}_{{\boldsymbol{ij}}}|)-{\boldsymbol{\gamma }}{{\boldsymbol{D}}}_{{\boldsymbol{ij}}},$$

where ***γ*** is a decay rate of the tube and ***f***(***Q***) is usually a simply increasing function with ***f***(**0**) = **0**^43^. The tubes without flux are removed, and the pressure at each node is updated during iterations. This process is repeated until the optimal path is found, thus indicating that as the brain information flow through the path between two nodes increases, the importance of that route increases. Finally, unused paths are removed in the *Physarum* model. There is a negative correlation between the length of the path and the amount of flux through the path.

#### Centrality measure

The criticality (***c***) of each edge ***e***_***ij***_ is defined as,

4 $${{\boldsymbol{c}}}_{{\boldsymbol{ij}}}=\sum _{{\boldsymbol{k}}}\,{{\boldsymbol{Q}}}_{{\boldsymbol{ij}}}^{{\boldsymbol{k}}},\,\,{\boldsymbol{k}}={\bf{1}},{\bf{2}},{\bf{3}},\,\ldots ,\,\frac{{\boldsymbol{n}}({\boldsymbol{n}}-{\bf{1}})}{{\bf{2}}},$$

where ${{\boldsymbol{Q}}}_{{\boldsymbol{ij}}}^{{\boldsymbol{k}}}$ is the ***k***th final flux through edge ***e***_***ij***_, and ***k*** are the different path indices between all different pairs of nodes, and ***c***_***ij***_ indicates the sum of the flux through the edge ***e***_***ij***_ between all pairs of nodes ***i*** and ***j***. Correspondingly, ***C***_***P***,***node***_of node ***i*** is defined as,

5 $${{\boldsymbol{C}}}_{{\boldsymbol{P}},{\boldsymbol{node}}}({\boldsymbol{i}})=\sum _{{\boldsymbol{j}}}\,{{\boldsymbol{c}}}_{{\boldsymbol{ij}}},$$

where ***c***_***ij***_ is the criticality (***c***) of each edge ***e***_***ij***_. In addition, ***C***_***P***,***node***_ is defined as the sum of the criticality of each edge ***e***_***ij***_ attached to ***i***. ***C***_***P***,***edge***_ was also calculated by the sum of flux (***c***) that passed through a given edge from the *Physarum* model.

### Other centrality measures

In this study, the values of ***C***_***D***,***node***_ and ***C***_***B***,***node***_ were compared with ***C***_***P***,***node***_. Equivalently, the value of ***C***_***D***,***node***_^28^ of node ***i*** is defined as,

6 $${{\boldsymbol{C}}}_{{\boldsymbol{D}},{\boldsymbol{node}}}({\boldsymbol{i}})=\sum _{{\boldsymbol{j}}}\,{{\boldsymbol{M}}}_{{\boldsymbol{ij}}},$$

where the ***C***_***D***,***node***_ of node ***i*** is given by the column sum of the connectivity matrix ***M***. In addition, ***C***_***D***,***node***_(***i***) captures the number of all edges connected to node ***i***, and ***C***_***B***,***node***_^3,8^ of node ***i*** is defined as,

7 $${{\boldsymbol{C}}}_{{\boldsymbol{B}},{\boldsymbol{node}}}({\boldsymbol{i}})=\sum _{{\boldsymbol{j}}\ne {\boldsymbol{i}}\ne {\boldsymbol{k}}}\frac{{{\boldsymbol{\rho }}}_{{\boldsymbol{jk}}}({\boldsymbol{i}})}{{{\boldsymbol{\rho }}}_{{\boldsymbol{jk}}}},$$

where ***ρ***_***jk***_ is the number of the shortest paths from node ***j*** to ***k***, and ***ρ***_***jk***_(***i***) is the number of the shortest paths between nodes ***j*** and ***k*** that pass through node ***i***. Accordingly, ***C***_***B***,***node***_(***i***) captures the influence of a node on the information flow between other nodes in the network. A node with a high degree of ***C***_***B***,***node***_ indicates increased interconnectivity with other regions in the network. Thus, the value of ***C***_***B***,***edge***_ was calculated as the number of the shortest paths that passes through a given edge instead of a node^33^. These measures were calculated using the Brain Connectivity Toolbox (http://www.brain-connectivity-toolbox.net).

### Statistical analyses

The Jaccard index (***J***) values of each set of network hubs or bridges from different centrality measures Were calculated to show how they overlapped quantitatively^79^. ***J*** was defined as the ratio of the number of overlapping network hubs or bridges to the total number of these hubs (or bridges) based on any two centrality measures,

8 $${\boldsymbol{J}}({\boldsymbol{A}},{\boldsymbol{B}})=\frac{|{\boldsymbol{A}}\cap {\boldsymbol{B}}|}{|{\boldsymbol{A}}\cup {\boldsymbol{B}}|}=\frac{|{\boldsymbol{A}}\cap {\boldsymbol{B}}|}{|{\boldsymbol{A}}|+|{\boldsymbol{B}}|-|{\boldsymbol{A}}\cap {\boldsymbol{B}}|},$$

where ***A*** and ***B*** are the sets of the hubs or bridges from each centrality measure, $|{\boldsymbol{A}}\cap {\boldsymbol{B}}|$ is the number of overlapping hubs or bridges, and $|{\boldsymbol{A}}\cup {\boldsymbol{B}}|$ is the total number of ***A*** and ***B*** hub or bridge sets. The value of ***J*** varies from zero (no overlap) to one (perfect overlap). Linear regression analyses was performed at all 78 network nodes to assess the relationship of three different pairs **(*C***_***D***,***node***_vs. ***C***_***B***,***node***_, ***C***_***P***,***node***_vs. ***C***_***D***,***node***_, and ***C***_***P***,***node***_ vs. ***C***_***B***,***node***_**)** in a continuous manner.

The Z-transform was applied on the centrality measures to ensure a fair comparison, and an ANOVA test was then performed to determine significant differences among centrality measures (***C***_***D***,***node***_, ***C***_***B***,***node***_, and ***C***_***P***,***node***_) from the different concepts. All 78 network nodes were analyzed separately, and P values such that P < 0.05 were considered statistically significant with Bonferroni post-hoc correction. Intersubject variability, which assesses whether centrality measures could be reliably reproduced across all subjects, was quantified based on the coefficient of variation (***CV***),

9 $${\boldsymbol{CV}}=\frac{{\boldsymbol{\sigma }}({{\boldsymbol{X}}}_{{\boldsymbol{n}}})}{{\boldsymbol{\mu }}({{\boldsymbol{X}}}_{{\boldsymbol{n}}})},\,{\boldsymbol{n}}={\bf{1}},{\bf{2}},{\bf{3}},\,\ldots ,\,{\boldsymbol{N}},$$

where ***X***_***n***_ is the normalized centrality value of a network node at the ***n***th subject, ***N*** is the total number of datasets, and σ and μ denote the standard deviation and mean, respectively. Equivalently, ***CV*** quantifies the central tendency and variability of the samples. Therefore, a lower ***CV*** indicates lower intersubject variability and higher consistency across subjects in the group.

## Supplementary information

Supplementary Table S1
