# Supplementary material for: A Physarum Centrality Measure of the Human Brain Network
Source: Sci Rep. 2019 Apr 11;9:5907. doi: 10.1038/s41598-019-42322-7 (PMC6459855; doi:10.1038/s41598-019-42322-7)
Supplement: Supplementary file 1 — Supplementary Table S1 [file 41598_2019_42322_MOESM1_ESM.docx]

**A *Physarum* Centrality Measure of the Human Brain Network**

Hunki Kwon^1,2^, Yong-Ho Choi^1^, Jong-Min Lee^1*^

^1^Department of Biomedical Engineering, Hanyang University, Seoul, South Korea

^2^Department of Neurology, Yale University School of Medicine, New Haven, Connecticut, USA

***Corresponding author:**

Jong-Min Lee
Department of Biomedical Engineering, Hanyang University
Sanhak-kisulkwan #319, 222 Wangsipri-ro, Sungdong-gu, Seoul, 04673, KOREA
Tel: +82-2-2220-0685
E-mail: [ljm@hanyang.ac.kr](mailto:ljm@hanyang.ac.kr)

**Supplementary Table S1**

**Table S1. Abbreviations for the cortical regions.**

| **Abbreviation** | **Automated anatomical labeling regions** |
| --- | --- |
| PreCG.L | Left precentral gyrus |
| PreCG.R | Right precentral gyrus |
| SFGdor.L | Left superior frontal gyrus, dorsolateral |
| SFGdor.R | Right superior frontal gyrus, dorsolateral |
| ORBsup.L | Left superior frontal gyrus, orbital part |
| ORBsup.R | Right superior frontal gyrus, orbital part |
| MFG.L | Left middle frontal gyrus |
| MFG.R | Right Middle frontal gyrus |
| ORBmid.L | Left middle frontal gyrus orbital part |
| ORBmid.R | Right middle frontal gyrus orbital part |
| IFGoperc.L | Left inferior frontal gyrus, opercular part |
| IFGoperc.R | Right inferior frontal gyrus, opercular part |
| IFGtriang.L | Left inferior frontal gyrus, triangular part |
| IFGtriang.R | Right inferior frontal gyrus, triangular part |
| ORBinf.L | Left inferior frontal gyrus, orbital part |
| ORBinf.R | Right inferior frontal gyrus, orbital part |
| ROL.L | Left Rolandic operculum |
| ROL.R | Right Rolandic operculum |
| SMA.L | Left supplementary motor area |
| SMA.R | Right supplementary motor area |
| OLF.L | Left olfactory cortex |
| OLF.R | Right olfactory cortex |
| SFGmed.L | Left superior frontal gyrus, medial |
| SFGmed.R | Right superior frontal gyrus, medial |
| ORBsupmed.L | Left superior frontal gyrus, medial orbital |
| ORBsupmed.R | Right superior frontal gyrus, medial orbital |
| REC.L | Left gyrus rectus |
| REC.R | Right gyrus rectus |
| INS.L | Left insula |
| INS.R | Right insula |
| ACG.L | Left anterior cingulate and paracingulate gyri |
| ACG.R | Right anterior cingulate and paracingulate gyri |
| DCG.L | Left median cingulate and paracingulate gyri |
| DCG.R | Right median cingulate and paracingulate gyri |
| PCG.L | Left posterior cingulate gyrus |
| PCG.R | Right posterior cingulate gyrus |
| PHG.L | Left parahippocampal gyrus |
| PHG.R | Right parahippocampal gyrus |
| CAL.L | Left calcarine fissure and surrounding cortex |
| CAL.R | Right calcarine fissure and surrounding cortex |
| CUN.L | Left cuneus |
| CUN.R | Right cuneus |
| LING.L | Left lingual gyrus |
| LING.R | Right lingual gyrus |
| SOG.L | Left superior occipital gyrus |
| SOG.R | Right superior occipital gyrus |
| MOG.L | Left middle occipital gyrus |
| MOG.R | Right Middle occipital gyrus |
| IOG.L | Left inferior occipital gyrus |
| IOG.R | Right inferior occipital gyrus |
| FFG.L | Left fusiform gyrus |
| FFG.R | Right fusiform gyrus |
| PoCG.L | Left postcentral gyrus |
| PoCG.R | Right postcentral gyrus |
| SPG.L | Left superior parietal gyrus |
| SPG.R | Right superior parietal gyrus |
| IPL.L | Left inferior parietal |
| IPL.R | Right inferior parietal |
| SMG.L | Left supramarginal gyrus |
| SMG.R | Right supramarginal gyrus |
| ANG.L | Left angular gyrus |
| ANG.R | Right angular gyrus |
| PCUN.L | Left precuneus |
| PCUN.R | Right precuneus |
| PCL.L | Left paracentral lobule |
| PCL.R | Right paracentral lobule |
| HES.L | Left Heschl gyrus |
| HES.R | Right Heschl gyrus |
| STG.L | Left superior temporal gyrus |
| STG.R | Right superior temporal gyrus |
| TPOsup.L | Left temporal pole: superior temporal gyrus |
| TPOsup.R | Right temporal pole: superior temporal gyrus |
| MTG.L | Left middle temporal gyrus |
| MTG.R | Right middle temporal gyrus |
| TPOmid.L | Left temporal pole: middle temporal gyrus |
| TPOmid.R | Right temporal pole: middle temporal gyrus |
| ITG.L | Left inferior temporal gyrus |
| ITG.R | Right inferior temporal gyrus |
